# Early‐Life Heat Stress Impairs Cognition and Alters Its Covariation With Behaviour in Zebrafish

**DOI:** 10.1002/ece3.72139

**Published:** 2025-09-11

**Authors:** Elia Gatto, Francesca Conti, Cristiano Bertolucci, Tyrone Lucon‐Xiccato

**Affiliations:** ^1^ Department of Chemical, Pharmaceutical and Agricultural Sciences University of Ferrara Ferrara Italy; ^2^ Department of Life Sciences and Biotechnology University of Ferrara Ferrara Italy

**Keywords:** climate change, cognitive plasticity, conservation behaviour, *Danio rerio*, global warming

## Abstract

As global temperatures rise, animals are increasingly exposed to heat stress, with growing evidence suggesting that such stress can impair cognitive functions. While research has documented these cognitive effects in adult mammals, birds and fish, less is known about the consequences of thermal stress during early development. Here, we investigated the effects of short‐term heat stress on learning in zebrafish ( *Danio rerio* ). Embryos were exposed to elevated temperatures (+5°C) for 2 days, beginning 10 days after fertilisation, and tested for learning performance 2 days post‐treatment. Heat‐stressed individuals showed reduced learning ability compared to controls. Furthermore, heat stress affected the covariation between learning and behavioural responses: in the group exposed to heat stress, individuals with stronger responses to stimulation learned faster, a relationship absent in control subjects. These results suggest that heat stress not only impairs cognitive performance but also alters the structure of behavioural‐cognitive trait associations. Given the ecological importance of cognition for survival and fitness, such changes may have far‐reaching consequences for wild populations.

## Graphical abstract

This study investigates how short‐term heat stress during early development affects cognition in zebrafish (*Danio rerio*) larvae. Embryos exposed to heat stress condition showed reduced learning performance compared to the control condition. Second, we found that heat stress alter the behavioural‐cognitive covariant structure, suggesting potential detrimental effect of fitness and survival.

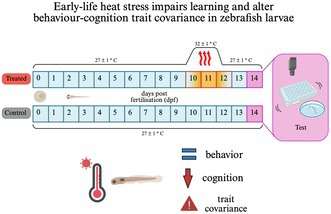

## 1 Introduction

With the intensification of climate change, prolonged periods of abnormally high temperatures are becoming increasingly frequent, severe and widespread across ecosystems (Perkins‐Kirkpatrick and Lewis 2020), including aquatic ones (Oliver et al. 2018, 2021). While the physiological and ecological impacts of extreme heat on animals are well established (reviewed in Smale et al. 2019; Smith et al. 2023; Wang et al. 2024), growing attention is now being directed toward the neurological and cognitive consequences of thermal stress. Indeed, the central nervous system is directly sensitive to external factors, including temperature (Taylor et al. 2016), as well as to physiological imbalances resulting from stressors (Maille and Schradin 2017). Studies on human populations have revealed that heatwaves can severely impair cognitive function (Laurent et al. 2018; Zhou et al. 2023; Zhu et al. 2022). It has been proposed that other species may also experience similar cognitive impairments due to increased heat stress, even over short periods (Soravia et al. 2021). Empirical data support this hypothesis, revealing significant negative effects of heat stress on cognition in a range of bird species (Blackburn et al. 2022; Danner et al. 2021; Soravia et al. 2023, 2024).

The impact of heat stress may be even greater in fish. This vertebrate group exhibits high cognitive plasticity, both at the phenotypic level (Bisazza and Lucon‐Xiccato 2025; Lucon‐Xiccato, De Russi, et al. 2023; Lucon‐Xiccato, Montalbano, and Bertolucci 2023) and at the cellular level (reviewed in Zupanc 2021). Moreover, as primarily ectothermic organisms, fish lack internal mechanisms to regulate their body temperature. Consequently, their physiological and neural functions are closely linked to environmental conditions. Studies in fish have shown that cognitive performance varies significantly depending on the temperature to which individuals are exposed (Babkiewicz et al. 2021; Maffioli et al. 2022; Silveira et al. 2023; Toni et al. 2019; but see: Guzman et al. 2023). Notably, one study found that even short‐term heat stress comparable in duration and intensity to heatwaves associated with climate change can impair cognition (Pereira et al. 2025).

In this study, we investigated the effects of short‐term heat stress in zebrafish ( *Danio rerio* ), focusing on early developmental stages. The zebrafish offer several advantages for our study due to its distinctive developmental biology. The embryonic development is rapid for a vertebrate, with hatching occurring approximately 3–4 days after fertilisation. The larvae remain relatively inactive for the first day post‐hatching, but by around 5 days post‐fertilisation, they begin to swim actively and feed independently (Lindsey et al. 2010), allowing behavioural measurements. This fast and well‐characterised developmental timeline makes the zebrafish a valuable species for conducting experiments on the developmental effects of environmental stressors (Eachus et al. 2021). Previous studies have demonstrated the sensitivity of zebrafish to heat stress from multiple perspectives (reviewed in López‐Olmeda and Sánchez‐Vázquez 2011). For instance, heat stress alters the expression of several genes, including those in the brain (Long et al. 2012; Nonnis et al. 2021), and reduces the energy available for metabolism, thereby limiting growth (Aguilar et al. 2022). It also affects locomotor activity, such as turning angle and immobility (Abozaid et al. 2020), and alters behaviours including exploration (Nonnis et al. 2021) and shoaling (Toni et al. 2025). Moreover, high temperatures can interact with other environmental stressors, such as increasing the toxicity of waterborne chemicals (Haapanen‐Saaristo et al. 2024). Cognitive studies show that zebrafish exposed to high temperatures have impaired recognition of previously visited locations when tested at 34°C versus 26°C (Maffioli et al. 2022; Toni et al. 2019). Interestingly, in another study, zebrafish exposed to higher temperatures (31°C vs. 21°C) appeared to learn more quickly to locate a food reward but also approached it more directly and frequently, potentially due to enhanced learning or increased motivation driven by metabolic demand (Babkiewicz et al. 2021). Notably, all of these cognitive studies focused on acute or chronic temperature increases during adulthood. To our knowledge, no studies in zebrafish have investigated the effects of short‐term heat stress, such as heatwaves, during early development, a critical period when the nervous system is particularly sensitive to environmental influences (Andreassen et al. 2022; Gatto et al. 2022; Groneberg et al. 2020).

In our experiment, we exposed embryos to heat stress (+5°C) for 2 days, starting 10 days after fertilisation, and assessed their cognitive abilities in a habituation learning test 2 days after the treatment ended. Based on previous research in fish and other taxa (e.g., Blackburn et al. 2022; Danner et al. 2021; Pereira et al. 2025; Soravia et al. 2023, 2024), we predicted a decline in cognitive performance in individuals exposed to heat stress. Additionally, we examined whether heat stress affects the covariation between cognitive and behavioural traits. Numerous studies have shown that individual fish differ in their cognitive abilities (reviewed in Lucon‐Xiccato and Bisazza 2017), and that this variation is often associated with consistent differences in behavioural traits (De Russi et al. 2024; Dugatkin and Alfieri 2003; Savaşçı et al. 2021; Trompf and Brown 2014; White et al. 2017). Environmental stressors have also been shown to alter the strength and structure of these covariations (Lucon‐Xiccato, De Russi, et al. 2023; Lucon‐Xiccato, Montalbano, and Bertolucci 2023). We therefore predicted that heat stress may affect how cognitive individual differences covary with behaviour.

## 2 Materials and Methods

### 2.1 Experimental Subjects

All experiments were conducted in accordance with the ASAB/ABS Guidelines for the Use of Animals in Research (Buchanan et al. 2012). The experimental protocols adopted in this research have been revised and approved by the Institutional Animal Care and Use Committee of the University of Ferrara (OPBA UNIFE, auth. n. TLX‐2022_1). At the end of the treatment and experimental observations, all surviving subjects were moved to maintenance tanks.

### 2.2 Experimental Subjects

Zebrafish larvae were obtained by natural spawning of wild‐type (‘Ariosto’ strain) adult breeders derived from fish bought from a local shop and maintained at the facility of University of Ferrara (Italy) since 2011. Juvenile and adult zebrafish were maintained in mixed‐sex groups of approximately 30 individuals (sex ratio 50:50) housed in 230‐L glass tanks provided with mechanical and biological filters. Water temperature was kept at 27°C ± 1°C, and chemical parameters were kept according to FELASA guidelines (pH 7.5 ± 1.0; conductivity < 500 μS/cm, NO_2_^−^ < 0.1 mg/L, NO_3_^−^ < 25 mg/L; Aleström et al. 2020). Fish were maintained under a LD 14:10 h (light/dark cycle) and daily fed three times with live *Artemia salina* nauplii ( *Artemia Salina* Premium GLS, Essen, Belgium) and commercial dry flake food (Vipan Nature, Sera, Heinsberg, Germany).

To obtain embryos, we placed breeding boxes into four maintenance tanks overnight. The breeding boxes were placed 5 cm below the water level and presented a grid bottom. The following morning (approximately 2 h after lights on), we collected eggs that had fallen below the grid and transferred them into Petri dishes (Ø10 cm, 1.5‐cm high) at a density (~35 eggs/Petri) based on the standard zebrafish husbandry guidelines (Aleström et al. 2020) and equal between the two treatments. The Petri dishes were filled with 30 mL E3 1× medium (5 mM NaCl, 0.17 mM KCl, 0.33 mM CaCl_2_, 0.33 mM MgSO_4_; Westerfield 2007) with the addition of 0.1 mL of antifungal agent methylene blue (0.0016 g/L, 0.0001%). As each tank hosted numerous adult zebrafish, the eggs collected were likely the result of multiple spawns from several individuals.

### 2.3 Experimental Protocol

The experiment was designed to compare a group of zebrafish larvae raised under constant temperature conditions with a group exposed to a single heat stress involving a relatively elevated temperature increase (+5°C). This dichotomous design was chosen based on earlier studies with a similar scope (e.g., Spinks et al. 2019; Thoral et al. 2022) and was expected to provide sufficient power to detect potential effects.

**FIGURE 1:**
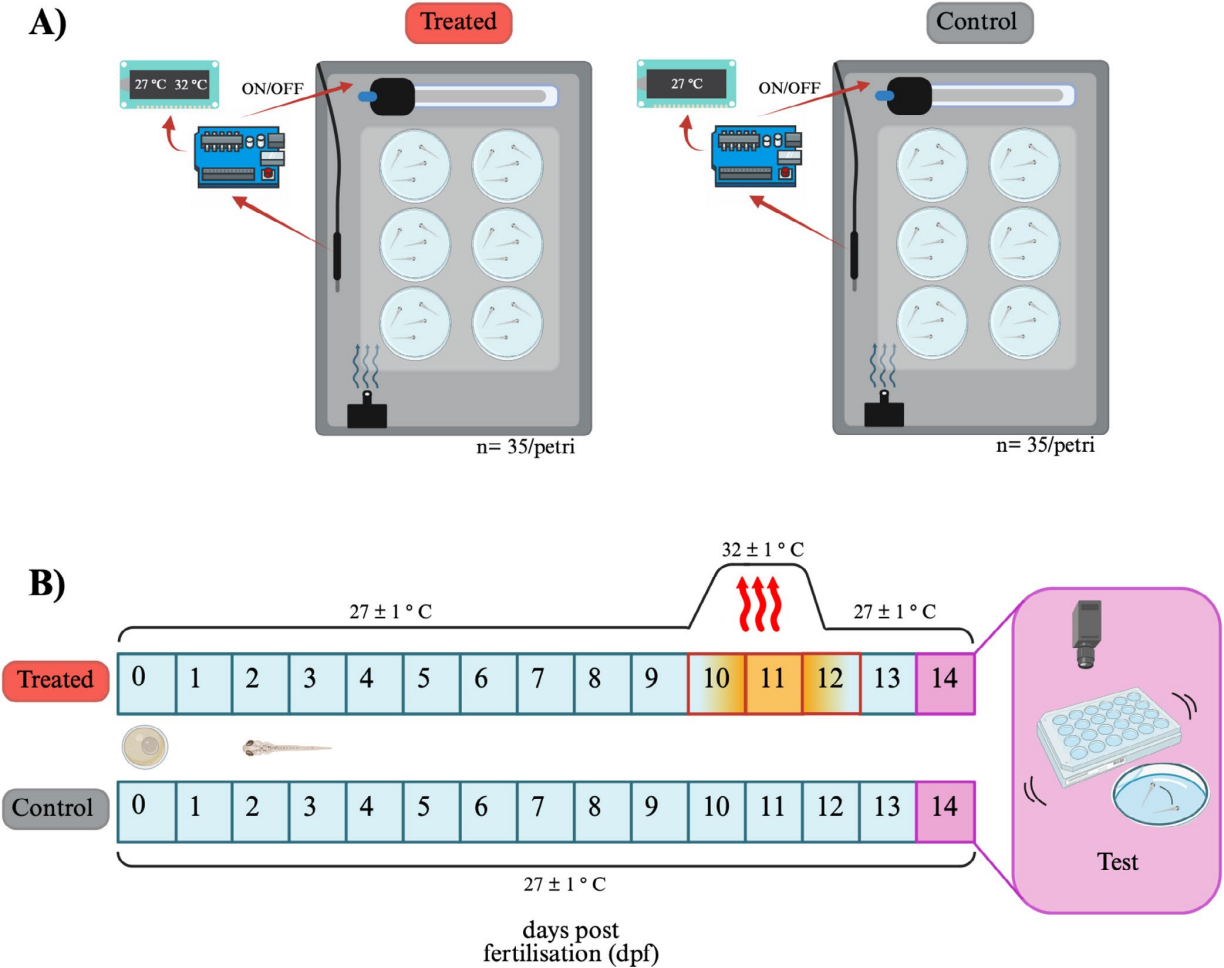
(A) Experimental set‐up used for the study. The eggs (0‐day post fertilisation, dpf) were placed in Petri dishes (6 Petri/condition; approximately 35 eggs/Petri) inside the experimental tanks. Identical boxes were to expose the subjects to the heat stress (left: treated group) or to the constat temperature (right: control group). The temperature was controlled by an automatic system consisting of a temperature probe, a heater and a microcontroller. (B) Schematic representation of the experimental protocol. From the start of experiment (0 dpf), larvae of both conditions were at 27°C ± 1°C until 10 dpf. Afterwards, the treated larvae undergo a two‐day heat‐stress (increase of 5°C, 10–12 dpf) while the control larvae remained at 27°C ± 1°C for the entire duration of the experiment. At 14 dpf, the larvae of both conditions were tested in the behavioural and cognitive assays.

After egg collection (0‐day post‐fertilisation, hereafter ‘dpf’; Westerfield 2007), the Petri dishes were kept under either the heat stress condition (hereafter, ‘treated’) or the control condition. For both conditions, the Petri dishes were transferred into 40 × 20 × 30 cm grey plastic boxes to avoid disturbance and maintain water temperature constant. The boxes were filled with water (10 cm) and the Petri dishes (6 Petri per condition; ~35 eggs/Petri) were placed on a transparent stand to keep them in contact with water without submerging them. A small pump (SunSun HJ‐311, China) recirculated the water to maintain thermal uniformity inside the boxes (Figure 1A). For both conditions, photoperiod was set to a LD 14:10 h cycle.

We used an automatic system to generate and control temperature based on a recently published work (Conti et al. 2025). Briefly, the temperature of the treatment box was controlled by an Arduino‐based microcontroller board (Elegoo Mega R3, Elegoo, China). The microcontroller was programmed to read the temperature data every 5 min from a probe (Ds18b20, AZ‐38 Delivery Vertriebs GmbH, Germany, tolerance ±1°C) submerged in the water (Figure 1A). The temperature value and time of acquisition were recorded on a microSD memory (SanDisk Ultra 32 GB, Milpitas, USA) and displayed on a 16 × 2 LCD Display (HD44780, AZ‐Delivery Vertriebs GmbH, 83 Germany), which allowed the experimenters to monitor temperature conditions live (Figure 1A). After reading the temperature value, the microcontroller switched on/off a heater (AquaHeat 50, 50 w, Juwel Aquarium, Germany) to maintain the inner temperature of the box according to a pre‐set threshold (Figure 1A).

In the treated condition, larvae were kept at 27°C (rearing temperature) from the beginning of the treatment (0 dpf) to 9 dpf (Figure 1B). In the morning of the 10th day (2 h after lights on), the internal temperature of the boxes was raised by one degree each hour up to 32°C (Thoral et al. 2022; Figure 1B). The heat stress was maintained for 2 days (10–12 dpf), then the temperature was decreased to the initial condition of 27°C by one degree every 90 min (Figure 1B). By using the same automatic system for maintaining and controlling the temperature, in the control condition water temperature was kept constant at 27°C ± 1°C throughout the experiment (Figure 1B).

The larvae were counted daily to annotate the mortality and then compare the survival probability of the two experimental groups at the end of treatment. After hatching (3 dpf), larvae showed swimming behaviour and started eating from the 5 dpf. Larvae were fed once a day with dry food (ZEBRAFEED, Sparos, Olhão, Portugal; particle size: 50–100 μm). During the daily check, we cleaned the Petri dish by removing uneaten food and faecal material and we refilled it with FishWater 1× solution (0.5 mM NaH_2_PO_4_·H_2_O, 0.5 mM Na_2_HPO_4_·H_2_O, 1.5 g Instant Ocean, 1 L deionised H_2_O).

We performed two replicates of the entire protocol described above by collecting eggs from two separate sets of spawns with a month interval (n° total sample of larvae at the start of experiment = 834; n° control larvae = 416; n° treated larvae = 418; n° of Petri dish per treatment = 12). The larvae from these treatments were used both to assess survival and to study behaviour and cognition.

### 2.4 Behavioural Assays

Before the cognitive test, we performed standard behavioural observations, which we aimed to use to study the covariation between cognitive and behavioural traits. These behavioural observations were performed when the subjects were 14 dpf. From the total sample of larvae that survived at the end of treatment (n° control larvae = 239; n° treated larvae = 240), we randomly selected 72 larvae from each experimental condition (n° total tested larvae = 144) to perform behavioural observations. We used the DanioVision Observation Chamber (Noldus, Wageningen, the Netherlands). In the first test, we assessed the behavioural responses of the larvae to a novel environment (open‐field test; Ahmad et al. 2012; Colwill and Creton 2011). Larvae from the two treatment groups were randomly placed in a 24‐well culture plate (12 larvae/condition per multi‐well plate; 6 replicates). Each well contained one individual larva and was filled with 1 mL of FishWater solution at 27°C. The general activity (distance covered, cm) of larvae was recorded for 60 min via EthovisionXT tracking software (ver. 11.5, Noldus). The second behavioural observation consisted of a vibrational stimulation administered with a solenoid (Tapping Device, Noldus, Wageningen, the Netherlands) controlled by the Ethovision XT software. The activity of the subjects was measured the second following the stimulation. This allowed us to measure the increase in swimming speed due to the response typical of the species (Fero et al. 2010).

### 2.5 Cognitive Assay

After the behavioural observations, the same subjects were exposed to a habituation learning test that allows cognitive assessment in early‐stage zebrafish larvae (Bellot et al. 2024; Faria et al. 2019; Gatto et al. 2024). The test consisted of administering 49 repeated vibration stimulations separated by 1 s via the solenoid. The 49 stimulations took place after the aforementioned initial stimulation used to infer the response of the subjects. Each repeated stimulation elicited a response consisting of an increase in swimming velocity. The habituation learning performance of each subject was assessed as the decrease in the response through the repeated stimulations (Faria et al. 2019).

The EthoVision software automatically tracked the position of subjects and calculated the distance covered for each second after the stimulation. To quantify the habituation learning score of each individual, we calculated a learning index based on the distance covered for each stimulation (i) as follows:

$$\;\text{learning}\;{\text{index}}_{\left[i\right]}=\frac{{\text{distance moved}}_{\left[i\right]}-{\text{distance moved}}_{\left[\text{first stimulation}\right]}}{{\text{distance moved}}_{\left[\text{first stimulation}\right]}}$$

The response to the first stimulus was expected to be the highest compared to the following responses. Lower values of the index (−1) indicated a reduction in individuals' response to the repeated stimulation, meaning a greater habituation learning ability.

### 2.6 Statistical Analysis

The statistical analyses were performed in RStudio version 4.2.1 (2022‐06‐23 ucrt) (Team RStudio 2019). Statistical tests were two‐tailed, and the significance level was set at *p* = 0.05. The difference in the survival probability between groups was assessed via the Kaplan–Meier method (Therneau 1997). The ‘survfit’ function created a survival curve for censored data using the Kaplan–Meier method (log‐rank method) based on the survival model created with the ‘Surv’ function (Therneau 2024). The survival probability was estimated by considering the number of living days of larvae (numeric value from 0 to 14) and their status at the end of the treatment (0 = dead, 1 = live).

We assessed behavioural and cognitive traits of 144 subjects, 72 control larvae and 72 treated larvae, a subsample of the treated larvae due to logistic constraints. However, inspection of video recordings revealed that 6 larvae did not move across the entire testing and were discarded. An additional 14 larvae were discarded as outliers after model inspection (response value was 10‐time more than Cook's distance threshold; Fox 2019). The final sample size for the behavioural and cognitive assays was 122 subjects (61 control and 61 treated larvae).

In the first behavioural test, that is, the open‐field test, differences in the general activity (response variable: distance covered, log‐transformed) between the two experimental groups were analysed with a linear mixed‐effects model (LMM, ‘lme4’ R package; Bates et al. 2015). As the predictors (fixed effects), we fitted treatment condition (treated vs. control) and time (sequence from 1 to 60 min time block, orthogonal polynomials settled via ‘contr.poly’ function from ‘stats’ R package, R Core Team 2021). The interaction term between these two fixed effects was included to assess differences in the temporal pattern of general activity between the two experimental groups. Individual identifier was fitted as a random effect to consider the repeated‐measure structure of the data. Significance of the model's parameters was assessed via Type II tests performed with ‘Anova’ from the car R package (Fox and Weisberg 2019). In the second behavioural test, that is, the vibrational stimulation, the response (log‐transformed distance covered) of subjects from the two experimental groups was compared with a two‐sample *t* test.

For the habituation learning test, we compared the temporal trend of the learning index between the experimental groups using a LMM (‘lme4’ R package; Bates et al. 2015). The model was fitted with the learning index as the response variable, and treatment condition and stimulation (sequential number from 1 to 50 stimulation settled as orthogonal polynomial via ‘contr.poly’ function from ‘stats’ R package) as fixed‐effect predictors. Moreover, the individual identifier was fitted as a random effect. We included the interaction term between treatment condition and stimulation to assess differences in the temporal pattern of response between the two experimental groups. When detecting a significant interaction term between the two fixed effects, we performed Tukey post hoc pairwise comparison using the ‘emmeans’ and ‘emtrends’ functions from ‘emmeans’ R packages, respectively (Lenth 2025).

To assess the difference in the trait covariation due to the heat stress, we performed Pearson correlation tests between behavioural and cognitive traits. We considered the overall activity (log‐transformed) measured in the open‐field test and the distance covered in response to the first stimulation (log‐transformed) as behavioural traits, while the average learning index (log‐transformed after excluding the response to the first stimulation) as a cognitive trait.

## 3 Results

### 3.1 Survival Rate

**FIGURE 2:**
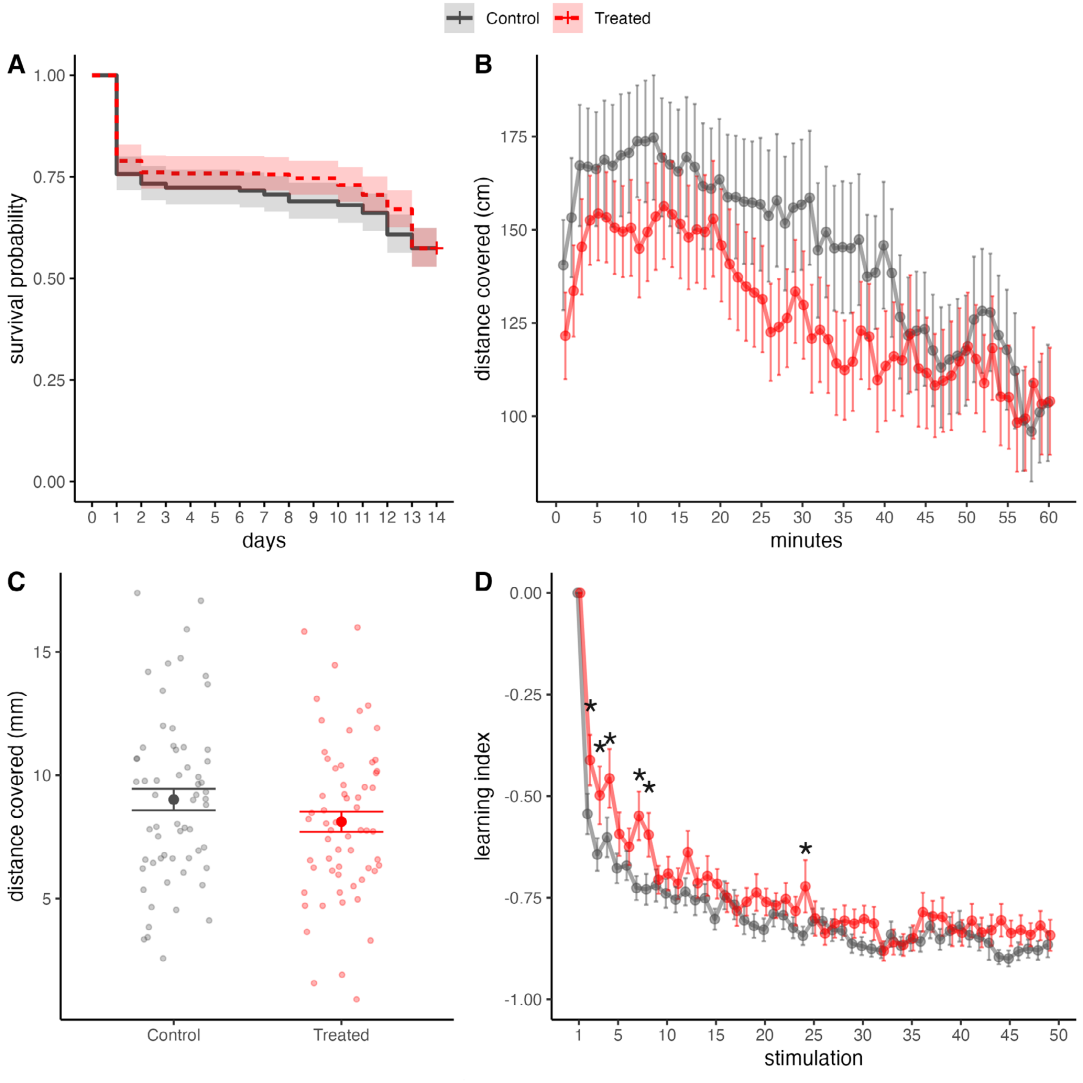
(A) Survival probability plot. (B) Temporal variation of distance covered in the open‐field test. (C) Response to the first vibrational stimulation measured as distance covered. (D) Temporal variation of the learning index in response to the repeated vibrational stimulations. To improve the clarity of the represented temporal pattern, the data point measured at the end of repeated stimulations (50th stimulation) was removed from the graph. Data are represented as mean ± standard error and divided for treated (red) and control condition (grey). Asterisks represent significant post hoc differences between conditions.

The analysis did not find a difference in the survival probability between the two experimental groups (n° control larvae = 416, n° survived control larvae = 239; n° heat treated larvae = 418, n° survived heat‐treated larvae = 240; *χ* ^2^_1_ = 0.100, *p* = 0.700; Figure 2A). This suggested that the heat stress did not affect the survival probability of the subjects.

### 3.2 Behavioural Assays

The general activity of larvae showed a significant decreasing trend over time (*χ* ^2^_59_ = 650.735, *p* < 0.001, linear trend: *t*_7080_ = −1.952, *p* < 0.001; Figure 2B). Overall, larvae from the two experimental groups did not differ in their activity (*χ* ^2^_1_ = 0.011, *p* = 0.915). The temporal decrease of activity did not differ between the two experimental groups (interaction treatment × time: *χ* ^2^_59_ = 47.940, *p* = 0.848).

In response to the first stimulation, the intensity of the response did not differ between the two experimental groups (activity of control group: 9.01 ± 3.42 mm; activity of treated group: 8.12 ± 3.20 mm; *t*_120_ = 1.501, *p* = 0.136; Figure 2C).

### 3.3 Habituation Learning Assay

Overall, the larvae showed a significant decrease of response across the stimulations (*χ* ^2^_49_ = 2580.534, *p* < 0.001; linear trend: estimate = −0.68 ± 0.03, *t*_5880_ = −23.901, *p* < 0.001; Figure 2D), which indicated learning. Compared to the treated group, the control groups showed a steeper decrease of the learning index (interaction treatment × stimulation: *χ* ^2^_49_ = 67.732, *p* = 0.039). Post hoc analysis revealed that the control group showed a greater habituation learning score compared to the treated group mostly in the earlier stimulations (pairwise comparisons: 2nd‐stimulation: *t*_695_ = −2.566, *p* = 0.015; 3rd‐stimulation: *t*_695_ = −2.819, *p* = 0.005; 4th‐stimulation: *t*_695_ = −2.813, *p* = 0.005; 7th‐stimulation: *t*_695_ = −3.442, *p* < 0.001; 8th‐stimulation: *t*_695_ = −2.600, *p* = 0.010; 24th‐stimulation: *t*_695_ = −2.357, *p* = 0.019; Figure 2D).

### 3.4 Covariations Analysis

**FIGURE 3:**
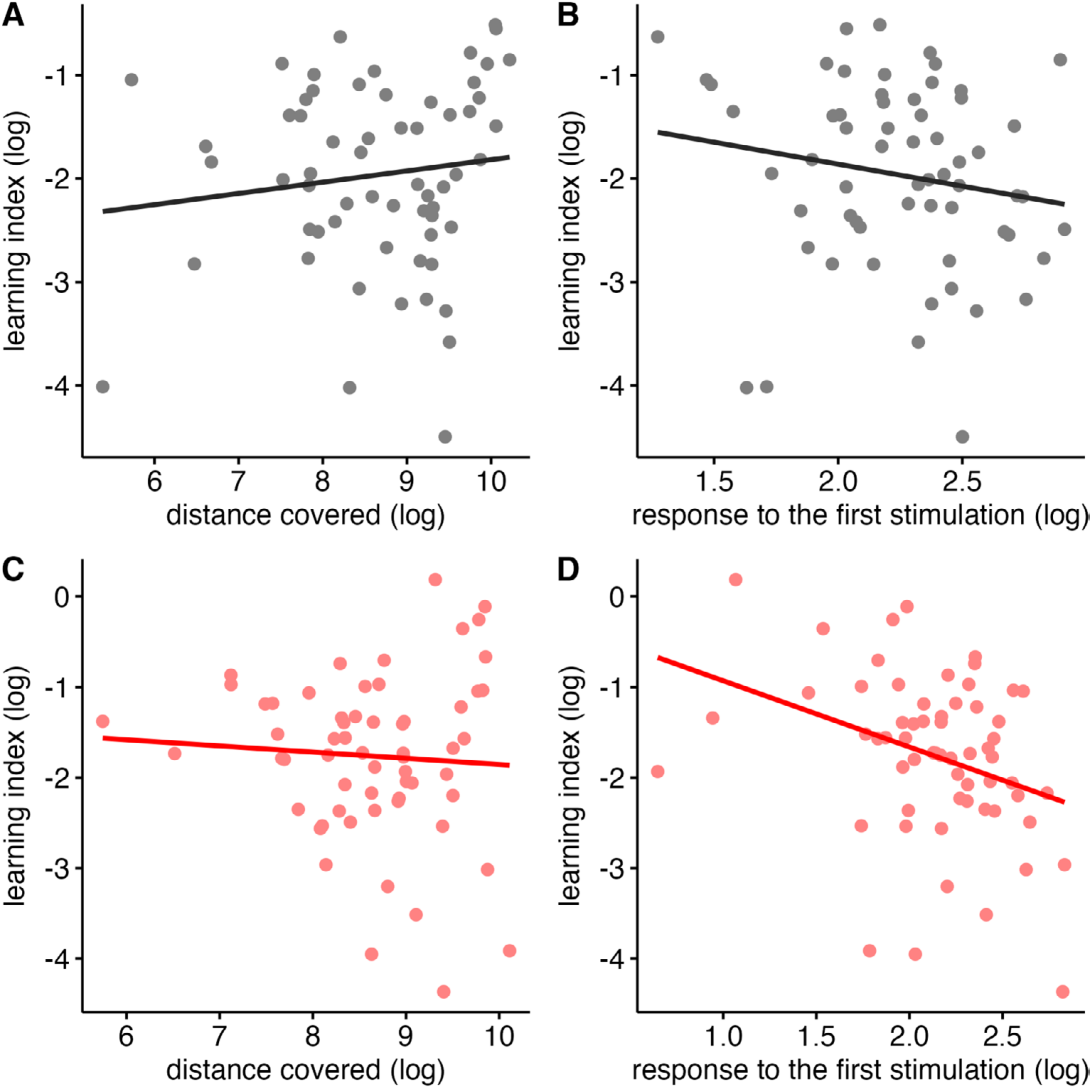
Scatterplots representing the covariation between the cognitive trait (learning index) and the two behavioural traits (A, C: Overall distance covered in the open‐field test; B, D: Locomotor response to the first vibrational stimulation) in the treated (red dots) and control group (grey dots). Lines represent the predicted linear relationship between traits. Behavioural and cognitive data were log‐transformed.

In the control group, the learning index did not correlate with the activity (Pearson *r* = 0.128, *t*_59_ = 0.993, *p* = 0.325; Figure 3A) and with the response to the first stimulation (Pearson *r* = −0.170, *t*_59_ = 1.324, *p* = 0.191; Figure 3B). In the treated group, however, the learning index significantly correlated with the response to the first stimulation (Pearson *r* = −0.335, *t*_59_ = −2.732, *p* = 0.008; Figure 3D), but not with the activity (Pearson *r* = −0.065, *t*_59_ = −0.498, *p* = 0.620; Figure 3C).

## 4 Discussion

The increasing heat associated with global warming is subjecting animals from various environments to escalating stress. This stress is not only necessarily lethal, but it potentially impacts animal fitness in less obvious ways. Growing literature in various species suggests a non‐lethal effect of heat stress may involve the cognitive system (Babkiewicz et al. 2021; Blackburn et al. 2022; Danner et al. 2021; Gérard et al. 2022; Pereira et al. 2025; Maffioli et al. 2022; Soravia et al. 2023, 2024; Toni et al. 2019). Based on this literature, we investigated the impact of early‐life heat stress on cognition in a teleost fish. Our findings suggest that learning abilities and their association with behaviour are affected after just 2 days of heat stress exposure in zebrafish larvae. This highlights that even brief exposure to thermal stress can influence the development of the cognitive and behavioural phenotype. Since cognition and behaviour play a key role in individuals' fitness, our findings suggest potentially detrimental effects on wild populations exposed to thermal stress.

The experimental heat stress was administered when the larvae were 7 days old, which corresponds to 10 days post‐fertilisation in this fast‐developing species. Colson et al. (2019) found that rainbow trout ( *Oncorhynchus mykiss* ), whose mothers were exposed to thermal stress, exhibited reduced spatial learning. Although their treatment duration was much longer than ours (6 weeks), their findings align with ours, suggesting that early development is a particularly sensitive window for heat stress affecting the cognitive systems of fish. This is especially concerning for species that lay eggs or display early stages with reduced mobility. While adult fish can partially avoid heat stress by moving to different water layers or habitats, larvae and eggs are often unable to migrate, leaving them more vulnerable to changes in environmental temperature.

In wild animals, cognition underpins critical survival behaviours such as foraging, predator avoidance, navigation and social interactions, all of which contribute significantly to individual fitness (Ashton et al. 2018; Cole et al. 2012; Smith et al. 2015). This holds true also for simple non‐associative learning forms shown by zebrafish larvae, such as habituation learning (Deecke et al. 2002; Dong and Clayton 2009; Geist 2011; Hemmi and Merkle 2009; Higham and Shelton 2011). We conclude that disruption of these cognitive functions due to elevated temperatures could have profound implications for both individual fitness and population dynamics.

We secondarily found that not only was the average learning performance affected by heat stress, but so were individual differences, particularly the correlation between learning and response to the stimulation. In the heat‐stressed group, individuals that exhibited stronger responses to stimulation also learned faster. This suggests that, in our learning test, which was based on a negative stimulus, shyer personalities may be more efficient learners, a pattern previously observed in guppies ( *Poecilia reticulata* ; Budaev and Zhuikov 1998). However, this correlation was absent in the control group of our experiment. Therefore, heat stress during development appeared to accentuate the covariation between cognitive and behavioural traits. While there is abundant evidence on how cognitive phenotypes affect fitness, we know far less about the ecological significance of the covariation between behaviour and cognition, an area of research that remains relatively young (Carere and Locurto 2011; Sih and Del Giudice 2012). Some evidence indicates that the presence and nature of these covariations in wild populations vary depending on environmental conditions, suggesting that they may result from local adaptations (Daniel and Bhat 2023), and that they are involved in activities critical for survival (De Russi et al. 2024). This implies that the heat‐stress‐induced changes we observed in zebrafish may negatively impact fitness by altering adaptive trait relationships. Alternatively, this strengthening of trait associations may resemble the one previously described between behavioural and metabolic traits (Killen et al. 2013), which have been linked to differences in sensitivity to stressors among behavioural and physiological phenotypes. Our study suggests that similar dynamics may exist between behaviour and cognition, though further research is required to confirm this.

In our study, we focused on a single thermal treatment and a single life stage as an initial investigation into the developmental effects of heat stress. However, there is a need to address these aspects more in detail. First, it is possible that the observed effect is modulated by the intensity of the thermal stress. Including treatments with both more and less extreme temperature increases may help clarify this point and provide a more comprehensive understanding of what may occur in natural environments. Similarly, prolonged exposure to heat stress could potentially worsen the impact, or alternatively, trigger developmental plasticity mechanisms that mitigate its negative effects (Scott and Johnston 2012). In addition, the duration of the observed cognitive alterations due to early exposure to heat stress should be investigated across life stages. We detected effects 2 days after the end of the heat treatment; however, we cannot exclude the possibility that they induce long‐lasting phenotypic modulation. Early‐life stressors are known to cause persistent plasticity in the neurobehavioural phenotype, including in zebrafish (Fontana et al. 2021; Gatto et al. 2022). If heat stress triggers a long‐term shift toward a particular cognitive phenotype, all the aforementioned fitness consequences may be amplified in natural environments. Greater attention should therefore be placed on early exposure to heat stress and its possible long‐term consequences throughout the animal's life.

## Author Contributions

**Elia Gatto:** conceptualization (equal), formal analysis (equal), funding acquisition (equal), investigation (equal), methodology (equal), software (lead), visualization (equal), writing – review and editing (equal). **Francesca Conti:** investigation (equal), methodology (equal), visualization (equal), writing – review and editing (equal). **Cristiano Bertolucci:** conceptualization (equal), funding acquisition (equal), methodology (equal), writing – review and editing (equal). **Tyrone Lucon‐Xiccato:** conceptualization (equal), funding acquisition (equal), methodology (equal), writing – original draft (lead), writing – review and editing (equal).

## Conflicts of Interest

The authors declare no conflicts of interest.

## Supporting information


**Data S1:** ece372139‐sup‐0001‐DataS1.xlsx.

## Data Availability

The datasets used for the analyses are provided as Supporting Information.

## References

[ece372139-bib-0001] Abozaid, A. , B. Tsang , and R. Gerlai . 2020. “The Effects of Small but Abrupt Change in Temperature on the Behavior of Larval Zebrafish.” Physiology & Behavior 227: 113169. 10.1016/j.physbeh.2020.113169. 32918940

[ece372139-bib-0002] Aguilar, A. , H. Mattos , B. Carnicero , et al. 2022. “Metabolomic Profiling Reveals Changes in Amino Acid and Energy Metabolism Pathways in Liver, Intestine and Brain of Zebrafish Exposed to Different Thermal Conditions.” Frontiers in Marine Science 9: 835379. 10.3389/fmars.2022.835379.

[ece372139-bib-0003] Ahmad, F. , L. P. Noldus , R. A. Tegelenbosch , and M. K. Richardson . 2012. “Zebrafish Embryos and Larvae in Behavioural Assays.” Behaviour 149: 1241–1281. 10.1163/1568539X-00003020.

[ece372139-bib-0004] Aleström, P. , L. D'Angelo , P. J. Midtlyng , et al. 2020. “Zebrafish: Housing and Husbandry Recommendations.” Laboratory Animals 54: 213–224. 10.1177/0023677219869037. 31510859 PMC7301644

[ece372139-bib-0005] Andreassen, A. H. , P. Hall , P. Khatibzadeh , F. Jutfelt , and F. Kermen . 2022. “Brain Dysfunction During Warming Is Linked to Oxygen Limitation in Larval Zebrafish.” Proceedings of the National Academy of Sciences of the United States of America 119: e2207052119. 10.1073/pnas.2207052119. 36122217 PMC9522358

[ece372139-bib-0006] Ashton, B. J. , A. R. Ridley , E. K. Edwards , and A. Thornton . 2018. “Cognitive Performance Is Linked to Group Size and Affects Fitness in Australian Magpies.” Nature 554: 364–367. 10.1038/nature25503. 29414945 PMC5815499

[ece372139-bib-0007] Babkiewicz, E. , K. Surga , Z. M. Gliwicz , and P. Maszczyk . 2021. “The Effect of Temperature on the Spatial Learning Rate of Zebrafish ( *Danio rerio* ).” Ethology 127: 632–642. 10.1111/eth.13197.

[ece372139-bib-0008] Bates, D. , M. Mächler , B. Bolker , and S. Walker . 2015. “Fitting Linear Mixed‐Effects Models Using lme4.” Journal of Statistical Software 67: 1–48. 10.18637/jss.v067.i01.

[ece372139-bib-0009] Bellot, M. , L. Manen‐Freixa , E. Prats , et al. 2024. “Short‐Term Exposure to Environmental Levels of Nicotine and Cotinine Impairs Visual Motor Response in Zebrafish Larvae Through a Similar Mode of Action: Exploring the Potential Role of Zebrafish α7 nAChR.” Science of the Total Environment 912: 169301. 10.1016/j.scitotenv.2023.169301. 38103609

[ece372139-bib-0010] Bisazza, A. , and T. Lucon‐Xiccato . 2025. “Individual Differences in Vertebrate Behavioural Lateralisation: The Role of Genes and Environment.” Symmetry 17: 527. 10.3390/sym17040527.

[ece372139-bib-0011] Blackburn, G. , E. Broom , B. J. Ashton , A. Thornton , and A. R. Ridley . 2022. “Heat Stress Inhibits Cognitive Performance in Wild Western Australian Magpies, *Cracticus tibicen dorsalis* .” Animal Behaviour 188: 1–11. 10.1016/j.anbehav.2022.03.016.

[ece372139-bib-0012] Buchanan, K. , T. Burt de Perera , C. Carere , et al. 2012. “Guidelines for the Treatment of Animals in Behavioural Research and Teaching.” Animal Behaviour 83: 301–309. 10.1016/j.anbehav.2011.10.031.

[ece372139-bib-0013] Budaev, S. V. , and A. Y. Zhuikov . 1998. “Avoidance Learning and “Personality” in the Guppy ( *Poecilia reticulata* ).” Journal of Comparative Psychology 112: 92–94. 10.1037/0735-7036.112.1.92.

[ece372139-bib-0014] Carere, C. , and C. Locurto . 2011. “Interaction Between Animal Personality and Animal Cognition.” Current Zoology 57: 491–498. 10.1093/czoolo/57.4.491.

[ece372139-bib-0015] Cole, E. F. , J. Morand‐Ferron , A. E. Hinks , and J. L. Quinn . 2012. “Cognitive Ability Influences Reproductive Life History Variation in the Wild.” Current Biology 22: 1808–1812. 10.1016/j.cub.2012.07.051. 22940473

[ece372139-bib-0016] Colson, V. , M. Cousture , D. Damasceno , et al. 2019. “Maternal Temperature Exposure Impairs Emotional and Cognitive Responses and Triggers Dysregulation of Neurodevelopment Genes in Fish.” PeerJ 7: e6338. 10.7717/peerj.6338. 30723624 PMC6360074

[ece372139-bib-0017] Colwill, R. M. , and R. Creton . 2011. “Locomotor Behaviors in Zebrafish ( *Danio rerio* ) Larvae.” Behavioural Processes 86: 222–229. 10.1016/j.beproc.2010.12.003. 21147203 PMC3063417

[ece372139-bib-0018] Conti, F. , G. de Alba , J. F. López‐Olmeda , et al. 2025. “An Automated Low‐Cost Solution for Creating a Multiple‐Step Thermal Gradient to Record Daily Fish Thermoregulatory Behavior.” Zebrafish 22: 15–19. 10.1089/zeb.2024.0153. 39711217

[ece372139-bib-0019] Daniel, D. K. , and A. Bhat . 2023. “Correlations Begin at Home: Drivers of Co‐Occurrence Patterns in Personality and Cognitive Ability in Wild Populations of Zebrafish.” Animal Cognition 26: 1381–1394. 10.1007/s10071-023-01787-w. 37248284

[ece372139-bib-0020] Danner, R. M. , C. M. Coomes , and E. P. Derryberry . 2021. “Simulated Heat Waves Reduce Cognitive and Motor Performance of an Endotherm.” Ecology and Evolution 11: 2261–2272. 10.1002/ece3.7194. 33717453 PMC7920763

[ece372139-bib-0021] De Russi, G. , M. Lanzoni , A. Bisazza , et al. 2024. “Eels' Individual Migratory Behavior Stems From a Complex Syndrome Involving Cognition, Behavior, Physiology, and Life History.” Proceedings of the National Academy of Sciences of the United States of America 121: e2407804121. 10.1073/pnas.2407804121. 39556736 PMC11621850

[ece372139-bib-0022] Deecke, V. B. , P. J. Slater , and J. K. Ford . 2002. “Selective Habituation Shapes Acoustic Predator Recognition in Harbour Seals.” Nature 420: 171–173. 10.1038/nature01030. 12432391

[ece372139-bib-0023] Dong, S. , and D. F. Clayton . 2009. “Habituation in Songbirds.” Neurobiology of Learning and Memory 92: 183–188. 10.1016/j.nlm.2008.09.009. 18845267 PMC2738998

[ece372139-bib-0024] Dugatkin, L. A. , and M. S. Alfieri . 2003. “Boldness, Behavioral Inhibition and Learning.” Ethology Ecology & Evolution 15: 43–49. 10.1080/08927014.2003.9522689.

[ece372139-bib-0025] Eachus, H. , M. K. Choi , and S. Ryu . 2021. “The Effects of Early Life Stress on the Brain and Behaviour: Insights From Zebrafish Models.” Frontiers in Cell and Developmental Biology 9: 657591. 10.3389/fcell.2021.657591. 34368117 PMC8335398

[ece372139-bib-0026] Faria, M. , E. Prats , K. A. Novoa‐Luna , et al. 2019. “Development of a Vibrational Startle Response Assay for Screening Environmental Pollutants and Drugs Impairing Predator Avoidance.” Science of the Total Environment 650: 87–96. 10.1016/j.scitotenv.2018.08.421. 30196226

[ece372139-bib-0027] Fero, K. , T. Yokogawa , and H. A. Burgess . 2010. “The Behavioral Repertoire of Larval Zebrafish.” In Zebrafish Models in Neurobehavioral Research, edited by A. Kalueff and J. Cachat , 249–291. Humana Press.

[ece372139-bib-0028] Fontana, B. D. , A. J. Gibbon , M. Cleal , W. H. Norton , and M. O. Parker . 2021. “Chronic Unpredictable Early‐Life Stress (CUELS) Protocol: Early‐Life Stress Changes Anxiety Levels of Adult Zebrafish.” Progress in Neuro‐Psychopharmacology and Biological Psychiatry 108: 110087. 10.1016/j.pnpbp.2020.110087. 32889032

[ece372139-bib-0029] Fox, J. 2019. Regression Diagnostics: An Introduction. Sage.

[ece372139-bib-0080] Fox, J. , and S. Weisberg . 2019. An R Companion to Applied Regression, Third Edition. Sage. https://www.john‐fox.ca/Companion/.

[ece372139-bib-0030] Gatto, E. , M. Dadda , M. Bruzzone , et al. 2022. “Environmental Enrichment Decreases Anxiety‐Like Behavior in Zebrafish Larvae.” Developmental Psychobiology 64: e22255. 10.1002/dev.22255. 35312057 PMC9313885

[ece372139-bib-0031] Gatto, E. , T. Lucon‐Xiccato , and C. Bertolucci . 2024. “Environmental Conditions Shape Learning in Larval Zebrafish.” Behavioural Processes 218: 105045. 10.1016/j.beproc.2024.105045. 38692461

[ece372139-bib-0032] Geist, V. 2011. “Wildlife Habituation: Advances in Understanding and Management Application.” Human‐Wildlife Interactions 5: 9–12.

[ece372139-bib-0033] Gérard, M. , A. Amiri , B. Cariou , and E. Baird . 2022. “Short‐Term Exposure to Heatwave‐Like Temperatures Affects Learning and Memory in Bumblebees.” Global Change Biology 28: 4251–4259. 10.1111/gcb.16196. 35429217 PMC9541601

[ece372139-bib-0034] Groneberg, A. H. , J. C. Marques , A. L. Martins , R. D. Del Corral , G. G. de Polavieja , and M. B. Orger . 2020. “Early‐Life Social Experience Shapes Social Avoidance Reactions in Larval Zebrafish.” Current Biology 30: 4009–4021. 10.1016/j.cub.2020.07.088. 32888479

[ece372139-bib-0035] Guzman, A. , O. Miller , and C. R. Gabor . 2023. “Elevated Water Temperature Initially Affects Reproduction and Behavior but Not Cognitive Performance or Physiology in *Gambusia affinis* .” General and Comparative Endocrinology 340: 114307. 10.1016/j.ygcen.2023.114307. 37172618

[ece372139-bib-0036] Haapanen‐Saaristo, A. M. , N. Virtanen , E. Tcarenkova , et al. 2024. “Heat Stress Sensitizes Zebrafish Embryos to Neurological and Cardiac Toxicity.” Biochemical and Biophysical Research Communications 733: 150682. 10.1016/j.bbrc.2024.150682. 39276696

[ece372139-bib-0037] Hemmi, J. M. , and T. Merkle . 2009. “High Stimulus Specificity Characterizes Anti‐Predator Habituation Under Natural Conditions.” Proceedings of the Royal Society B: Biological Sciences 276: 4381–4388. 10.1098/rspb.2009.1452. PMC2817115 19776070

[ece372139-bib-0038] Higham, J. E. S. , and E. J. Shelton . 2011. “Tourism and Wildlife Habituation: Reduced Population Fitness or Cessation of Impact?” Tourism Management 32: 1290–1298. 10.1016/j.tourman.2010.12.006.

[ece372139-bib-0039] Killen, S. S. , S. Marras , N. B. Metcalfe , D. J. McKenzie , and P. Domenici . 2013. “Environmental Stressors Alter Relationships Between Physiology and Behaviour.” Trends in Ecology & Evolution 28: 651–658. 10.1016/j.tree.2013.05.005. 23756106

[ece372139-bib-0040] Laurent, J. G. C. , A. Williams , Y. Oulhote , A. Zanobetti , J. G. Allen , and J. D. Spengler . 2018. “Reduced Cognitive Function During a Heat Wave Among Residents of Non‐Air‐Conditioned Buildings: An Observational Study of Young Adults in the Summer of 2016.” PLoS Medicine 15: e1002605. 10.1371/journal.pmed.1002605. 29990359 PMC6039003

[ece372139-bib-0041] Lenth, R. 2025. “emmeans: Estimated Marginal Means, aka Least‐Squares Means.” R package version 1.11.0‐001. https://rvlenth.github.io/emmeans/.

[ece372139-bib-0042] Lindsey, B. W. , F. M. Smith , and R. P. Croll . 2010. “From Inflation to Flotation: Contribution of the Swimbladder to Whole‐Body Density and Swimming Depth During Development of the Zebrafish ( *Danio rerio* ).” Zebrafish 7: 85–96. 10.1089/zeb.2009.0616. 20415646

[ece372139-bib-0043] Long, Y. , L. Li , Q. Li , X. He , and Z. Cui . 2012. “Transcriptomic Characterization of Temperature Stress Responses in Larval Zebrafish.” PLoS One 7: e37209. 10.1371/journal.pone.0037209. 22666345 PMC3364249

[ece372139-bib-0044] López‐Olmeda, J. F. , and F. J. Sánchez‐Vázquez . 2011. “Thermal Biology of Zebrafish ( *Danio rerio* ).” Journal of Thermal Biology 36: 91–104. 10.1016/j.jtherbio.2010.12.005.

[ece372139-bib-0045] Lucon‐Xiccato, T. , and A. Bisazza . 2017. “Individual Differences in Cognition Among Teleost Fishes.” Behavioural Processes 141: 184–195. 10.1016/j.beproc.2017.01.015. 28126389

[ece372139-bib-0046] Lucon‐Xiccato, T. , G. De Russi , S. Cannicci , E. Maggi , and C. Bertolucci . 2023. “Embryonic Exposure to Artificial Light at Night Impairs Learning Abilities and Their Covariance With Behavioural Traits in Teleost Fish.” Biology Letters 19: 20230436. 10.1098/rsbl.2023.0436. 37990566 PMC10663786

[ece372139-bib-0047] Lucon‐Xiccato, T. , G. Montalbano , and C. Bertolucci . 2023. “Adaptive Phenotypic Plasticity Induces Individual Variability Along a Cognitive Trade‐Off.” Proceedings of the Royal Society B: Biological Sciences 290: 20230350. 10.1098/rspb.2023.0350. PMC10291716 37357854

[ece372139-bib-0048] Maffioli, E. , E. Angiulli , S. Nonnis , et al. 2022. “Brain Proteome and Behavioural Analysis in Wild Type, BDNF+/− and BDNF−/− Adult Zebrafish (*Danio rerio*) Exposed to Two Different Temperatures.” International Journal of Molecular Sciences 23: 5606. 10.3390/ijms23105606. 35628418 PMC9146406

[ece372139-bib-0049] Maille, A. , and C. Schradin . 2017. “Ecophysiology of Cognition: How Do Environmentally Induced Changes in Physiology Affect Cognitive Performance?” Biological Reviews 92: 1101–1112. 10.1111/brv.12270. 27020603

[ece372139-bib-0050] Nonnis, S. , E. Angiulli , E. Maffioli , et al. 2021. “Acute Environmental Temperature Variation Affects Brain Protein Expression, Anxiety and Explorative Behaviour in Adult Zebrafish.” Scientific Reports 11: 2521. 10.1038/s41598-021-81804-5. 33510219 PMC7843641

[ece372139-bib-0051] Oliver, E. C. , J. A. Benthuysen , S. Darmaraki , et al. 2021. “Marine Heatwaves.” Annual Review of Marine Science 13: 313–342. 10.1146/annurev-marine-032720-095144. 32976730

[ece372139-bib-0052] Oliver, E. C. , M. G. Donat , M. T. Burrows , et al. 2018. “Longer and More Frequent Marine Heatwaves Over the Past Century.” Nature Communications 9: 1324. 10.1038/s41467-018-03732-9. PMC5893591 29636482

[ece372139-bib-0053] Pereira, B. , L. Cascalheira , R. Rosa , and J. R. Paula . 2025. “Alteration of Cleaner Wrasse Cognition and Brain Morphology Under Marine Heatwaves.” Functional Ecology 39: 1894–1905. 10.1111/1365-2435.70014.

[ece372139-bib-0054] Perkins‐Kirkpatrick, S. E. , and S. C. Lewis . 2020. “Increasing Trends in Regional Heatwaves.” Nature Communications 11: 3357. 10.1038/s41467-020-16970-7. PMC7334217 32620857

[ece372139-bib-0081] R Core Team . 2021. “R: A Language and Environment for Statistical Computing.” R Foundation for Statistical Computing, Vienna, Austria. https://www.R‐project.org/.

[ece372139-bib-0079] RStudio Team . 2019. “RStudio: Integrated Development for R.” RStudio, Inc., Boston, MA. http://www.rstudio.com/.

[ece372139-bib-0055] Savaşçı, B. B. , T. Lucon‐Xiccato , and A. Bisazza . 2021. “Ontogeny and Personality Affect Inhibitory Control in Guppies, *Poecilia reticulata* .” Animal Behaviour 180: 111–121. 10.1016/j.anbehav.2021.08.013.

[ece372139-bib-0056] Scott, G. R. , and I. A. Johnston . 2012. “Temperature During Embryonic Development Has Persistent Effects on Thermal Acclimation Capacity in Zebrafish.” Proceedings of the National Academy of Sciences of the United States of America 109: 14247–14252. 10.1073/pnas.120501210. 22891320 PMC3435178

[ece372139-bib-0057] Sih, A. , and M. Del Giudice . 2012. “Linking Behavioural Syndromes and Cognition: A Behavioural Ecology Perspective.” Philosophical Transactions of the Royal Society, B: Biological Sciences 367: 2762–2772. 10.1098/rstb.2012.0216. PMC3427552 22927575

[ece372139-bib-0058] Silveira, M. M. , J. M. Donelson , M. I. McCormick , H. Araujo‐Silva , and A. C. Luchiari . 2023. “Impact of Ocean Warming on a Coral Reef Fish Learning and Memory.” PeerJ 11: e15729. 10.7717/peerj.15729. 37576501 PMC10416774

[ece372139-bib-0059] Smale, D. A. , T. Wernberg , E. C. Oliver , et al. 2019. “Marine Heatwaves Threaten Global Biodiversity and the Provision of Ecosystem Services.” Nature Climate Change 9: 306–312. 10.1038/s41558-019-0412-1.

[ece372139-bib-0060] Smith, C. , A. Philips , and M. Reichard . 2015. “Cognitive Ability Is Heritable and Predicts the Success of an Alternative Mating Tactic.” Proceedings of the Royal Society B: Biological Sciences 282: 20151046. 10.1098/rspb.2015.1046. PMC4590461 26041347

[ece372139-bib-0061] Smith, K. E. , M. T. Burrows , A. J. Hobday , et al. 2023. “Biological Impacts of Marine Heatwaves.” Annual Review of Marine Science 15: 119–145. 10.1146/annurev-marine-032122-121437. 35977411

[ece372139-bib-0062] Soravia, C. , B. J. Ashton , A. Thornton , A. R. Bourne , and A. R. Ridley . 2024. “High Temperatures During Early Development Reduce Adult Cognitive Performance and Reproductive Success in a Wild Animal Population.” Science of the Total Environment 912: 169111. 10.1016/j.scitotenv.2023.169111. 38070557

[ece372139-bib-0063] Soravia, C. , B. J. Ashton , A. Thornton , and A. R. Ridley . 2021. “The Impacts of Heat Stress on Animal Cognition: Implications for Adaptation to a Changing Climate.” Wiley Interdisciplinary Reviews: Climate Change 12: e713. 10.1002/wcc.713.

[ece372139-bib-0064] Soravia, C. , B. J. Ashton , A. Thornton , and A. R. Ridley . 2023. “High Temperatures Are Associated With Reduced Cognitive Performance in Wild Southern Pied Babblers.” Proceedings of the Royal Society B 290: 20231077. 10.1098/rspb.2023.1077. 37989242 PMC10688443

[ece372139-bib-0065] Spinks, R. K. , P. L. Munday , and J. M. Donelson . 2019. “Developmental Effects of Heatwave Conditions on the Early Life Stages of a Coral Reef Fish.” Journal of Experimental Biology 222: jeb202713. 10.1242/jeb.202713. 31444281

[ece372139-bib-0066] Taylor, L. , S. L. Watkins , H. Marshall , B. J. Dascombe , and J. Foster . 2016. “The Impact of Different Environmental Conditions on Cognitive Function: A Focused Review.” Frontiers in Physiology 6: 372. 10.3389/fphys.2015.00372. 26779029 PMC4701920

[ece372139-bib-0067] Therneau, T. 2024. “A Package for Survival Analysis in R.” R package version 3.8‐3. https://CRAN.R‐project.org/package=survival.

[ece372139-bib-0068] Therneau, T. M. 1997. “Extending the Cox Model.” In Proceedings of the First Seattle Symposium in Biostatistics. Lecture Notes in Statistics 123, edited by D. Y. Lin and T. R. Fleming , 51–84. Springer. 10.1007/978-1-4684-6316-3_5.

[ece372139-bib-0069] Thoral, E. , D. Roussel , L. Quispe , Y. Voituron , and L. Teulier . 2022. “Absence of Mitochondrial Responses in Muscles of Zebrafish Exposed to Several Heat Waves.” Comparative Biochemistry and Physiology Part A: Molecular & Integrative Physiology 274: 111299. 10.1016/j.cbpa.2022.111299. 36031060

[ece372139-bib-0070] Toni, M. , E. Angiulli , G. Miccoli , et al. 2019. “Environmental Temperature Variation Affects Brain Protein Expression and Cognitive Abilities in Adult Zebrafish ( *Danio rerio* ): A Proteomic and Behavioural Study.” Journal of Proteomics 204: 103396. 10.1016/j.jprot.2019.103396. 31150779

[ece372139-bib-0071] Toni, M. , F. Frabetti , G. Tedeschi , and E. Alleva . 2025. “Effects of Environmental Temperature Variation on the Spatio‐Temporal Shoaling Behaviour of Adult Zebrafish (*Danio rerio*): A Two‐and Three‐Dimensional Analysis.” Animals 15: 2006. 10.3390/ani15142006. 40723469 PMC12291765

[ece372139-bib-0072] Trompf, L. , and C. Brown . 2014. “Personality Affects Learning and Trade‐Offs Between Private and Social Information in Guppies, *Poecilia reticulata* .” Animal Behaviour 88: 99–106. 10.1016/j.anbehav.2013.11.022.

[ece372139-bib-0073] Wang, J. H. , L. Zhang , P. J. Zhang , and B. J. Sun . 2024. “Physiological Processes Through Which Heatwaves Threaten Fauna Biodiversity.” Innovation Life 2: 100069. 10.59717/j.xinn-life.2024.100069.

[ece372139-bib-0074] Westerfield, M. 2007. The Zebrafish Book. A Guide for the Laboratory Use of Zebrafish ( *Danio rerio* ). Eugene University of Oregon Press.

[ece372139-bib-0075] White, S. L. , T. Wagner , C. Gowan , and V. A. Braithwaite . 2017. “Can Personality Predict Individual Differences in Brook Trout Spatial Learning Ability?” Behavioural Processes 141: 220–228. 10.1016/j.beproc.2016.08.009. 27567303

[ece372139-bib-0076] Zhou, W. , Q. Wang , R. Li , et al. 2023. “The Effects of Heatwave on Cognitive Impairment Among Older Adults: Exploring the Combined Effects of Air Pollution and Green Space.” Science of the Total Environment 904: 166534. 10.1016/j.scitotenv.2023.166534. 37647952

[ece372139-bib-0077] Zhu, H. , S. Hu , and C. W. Yu . 2022. “Cognitive Performance in a Warming Planet.” Indoor and Built Environment 31: 2195–2198. 10.1177/1420326X221116786.

[ece372139-bib-0078] Zupanc, G. K. 2021. “Adult Neurogenesis in the Central Nervous System of Teleost Fish: From Stem Cells to Function and Evolution.” Journal of Experimental Biology 224: jeb226357. 10.1242/jeb.226357. 33914040

